# A rare incidence of severe dermatological toxicities triggered by concomitant administration of all-trans retinoic acid and triazole antifungal in patients with acute promyelocytic leukemia: a case series and review of the literature

**DOI:** 10.1186/s13256-024-04577-1

**Published:** 2024-05-27

**Authors:** Aisha Jamal, Rafia Hassam, Qurratulain Rizvi, Ali Saleem, Anum Khalid, Nida Anwar

**Affiliations:** 1 Section of Malignant Haematology, National Institute of Blood Disease and Bone Marrow Transplantation, Karachi, Pakistan; 2 Research and Development, National Institute of Blood Disease and Bone Marrow Transplantation, Karachi, Pakistan; 3 Department of Clinical Haematology, National Institute of Blood Diseases and Bone Marrow Transplantation, Plot # Special D-3, Block-6, (Across Railway Line), P.E.C.H.S, Karachi, Pakistan

**Keywords:** Exfoliative dermatitis, Pharmacokinetic drug interaction, Necrotic scrotal ulceration, All-trans retinoic acid toxicity, Triazole anti-fungal

## Abstract

**Background:**

All-trans retinoic acid (ATRA) is an indispensable part of the treatment of acute promyelocytic leukemia (APL). Although, mild cutaneous toxicities like mucocutaneous xerosis, rash, and pruritus are well reported, ATRA associated severe dermatological toxicities are extremely rare. ATRA is primary metabolized by cytochrome P450 (CYP450) enzyme system, and triazole antifungals are notorious for their strong inhibitory effect on CYP450.

**Case presentation:**

Three Asian APL patients experienced rare ATRA-induced severe dermatological toxicities: exfoliative dermatitis (ED) in cases 1 and 2, and necrotic scrotal ulceration in case 3. Both case 1 (33-year-old female), and case 2 (28-year-old male) landed in emergency department with dehydration, generalized skin erythema and xerosis during their induction chemotherapy. Both of these patients also developed invasive aspergillosis and required concomitant triazole antifungals during their chemotherapy. For ED, intravenous fluids and broad-spectrum antibiotics were started along with application of local emollients to prevent transdermal water loss. Although their general condition improved but skin exfoliation continued with complete desquamation of palms and soles. Dermatology was consulted, and clinical diagnosis of ED was established. Discontinuation of ATRA resulted in complete resolution of ED. Case 3 (15-year-old boy) reported two blackish mildly tender scrotal lesions during induction chemotherapy. He also had mucocutaneous candidiasis at presentation and was kept on triazole antifungal. Local bacterial & fungal cultures, and serological testing for herpes simplex virus were reported negative. Despite adequate local care and optimal antibiotic support, his lesions persisted, and improved only after temporary discontinuation of ATRA. After a thorough literature review and considering the temporal association of cutaneous toxicities with triazole antifungals, we speculate that the concomitant use of triazole antifungals inhibited the hepatic metabolism of ATRA, resulting in higher serum ATRA concentration, and markedly accentuated cutaneous toxicities in our patients.

**Conclusion:**

By highlighting this crucial pharmacokinetic interaction, we want to caution the fellow oncologists to be mindful of the inhibitory effect of triazole antifungals on CYP450. We propose using a non-myelosuppressive combination of ATRA and arsenic trioxide for management of APL hence, obliterating the need of prophylactic antifungals. However, in the event of invasive fungal infection (IFI), we suggest using alternative class of antifungals.

## Background

Acute promyelocytic leukemia (APL) is a rare and potentially curable subtype of acute myeloid leukemia (AML), accounting for 5–8% of AML cases [1]. Genetically, APL is characterized by reciprocal translocation t(15:17) (q22;q11–12), with consequent fusion of promyelocytic (PML) gene on chromosome 15q22 to retinoic acid receptor-alpha (RAR-alpha) gene on chromosome 17q21. The resultant fusion oncoprotein, PML-RARA, induces transcriptional repression, chromatin condensation, maturation arrest, and accumulation of abnormal promyelocytes [2]. Advent of all-trans retinoic acid (ATRA) has revolutionized the treatment landscape of APL, and along with the backbone of anthracycline based chemotherapy, it is considered to be the standard of care for APL patients. Combination treatment with ATRA plus anthracycline based chemotherapy achieves an overall complete remission and cure rate of 95% and 80% respectively, rendering ATRA indispensable in the management of APL [3].

ATRA, an active metabolite of vitamin A, belongs to a class of retinoids. Although retinoids are well known for their dermatological side effects like xerosis, xerostomia, erythema, pruritis, and exfoliation; severe dermatological side effects of ATRA, especially in the dosage pertinent to APL (45 mg/m^2^), are rare. So far, only a single case of exfoliative dermatitis (ED) and a few cases of scrotal ulceration have been reported in literature [4–17]. We, here in, report a case series of three patients with serious and rare ATRA associated dermatological complications. We have also discussed upon the potentially precipitating pharmacokinetic interactions, as well as the detailed clinical course and management of our patients as simply withholding ATRA can jeopardize the outcome of this potentially curable malignant disorder.

## Case presentation

**Fig. 1:**
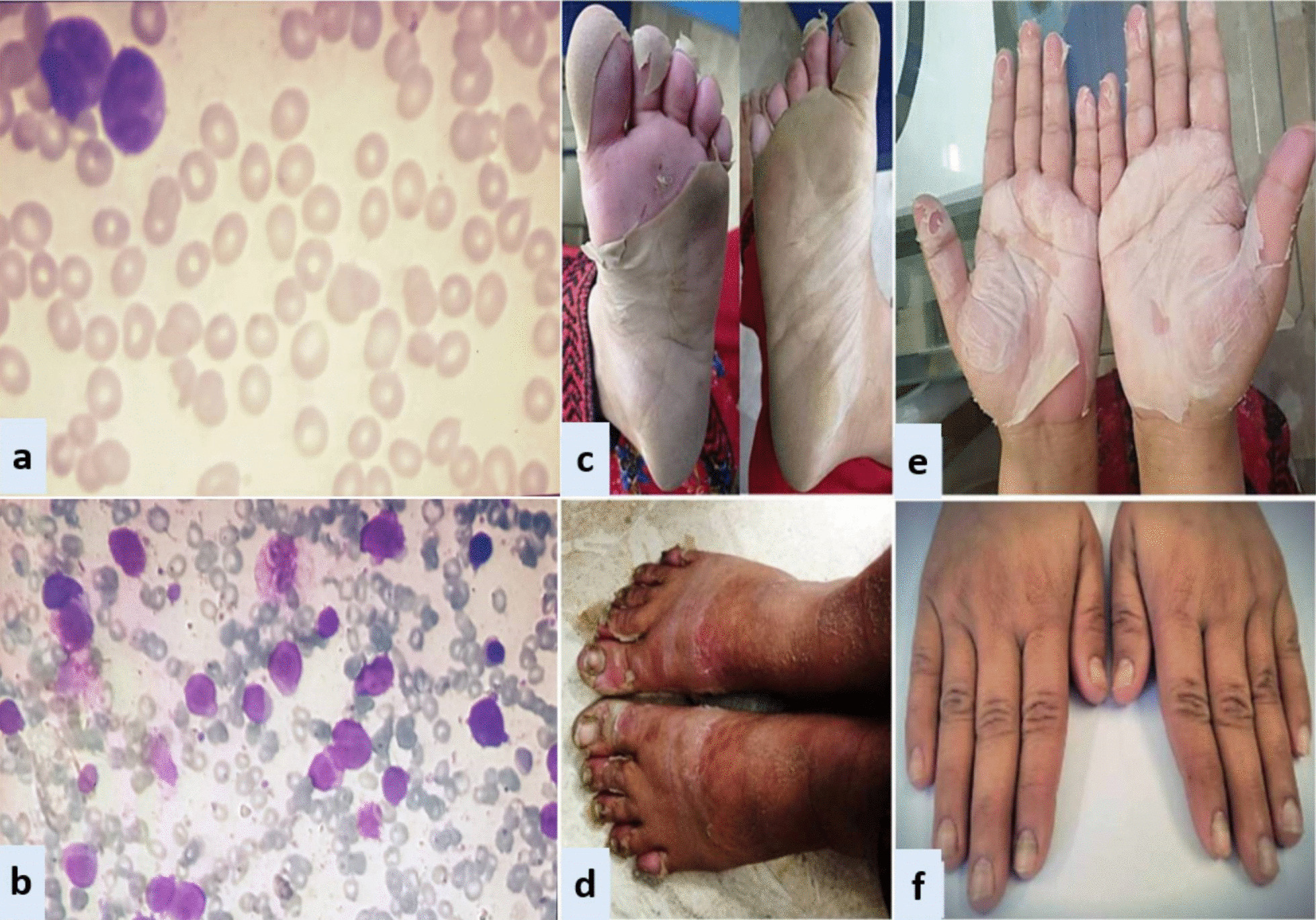
Exfoliative dermatitis &Onychomadesis (CASE 1). **a** Peripheral smear. **b** Bone marrow aspirate. **c** Desquamation of soles. **d** Desquamation of palms. **e** Dry exfoliation of feet and shins. **f** Onychomadesis

**Fig. 2:**
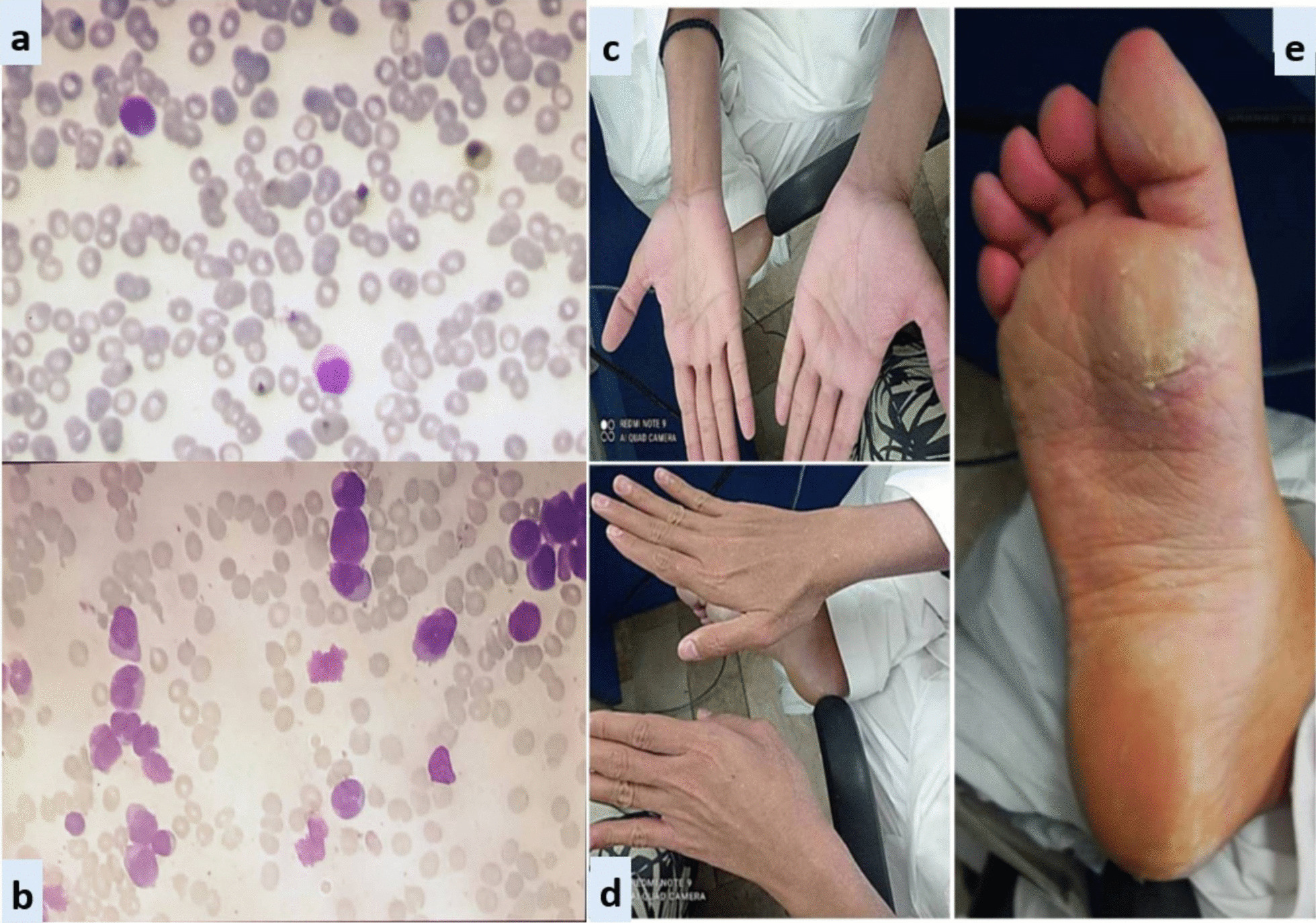
Exfoliative dermatitis (CASE 2). **a** Peripheral smear. **b** Bone marrow aspirate. **c** and **d** Erythema and scaling of hands. **e** Cutaneous desquamation of soles

**Fig. 3:**
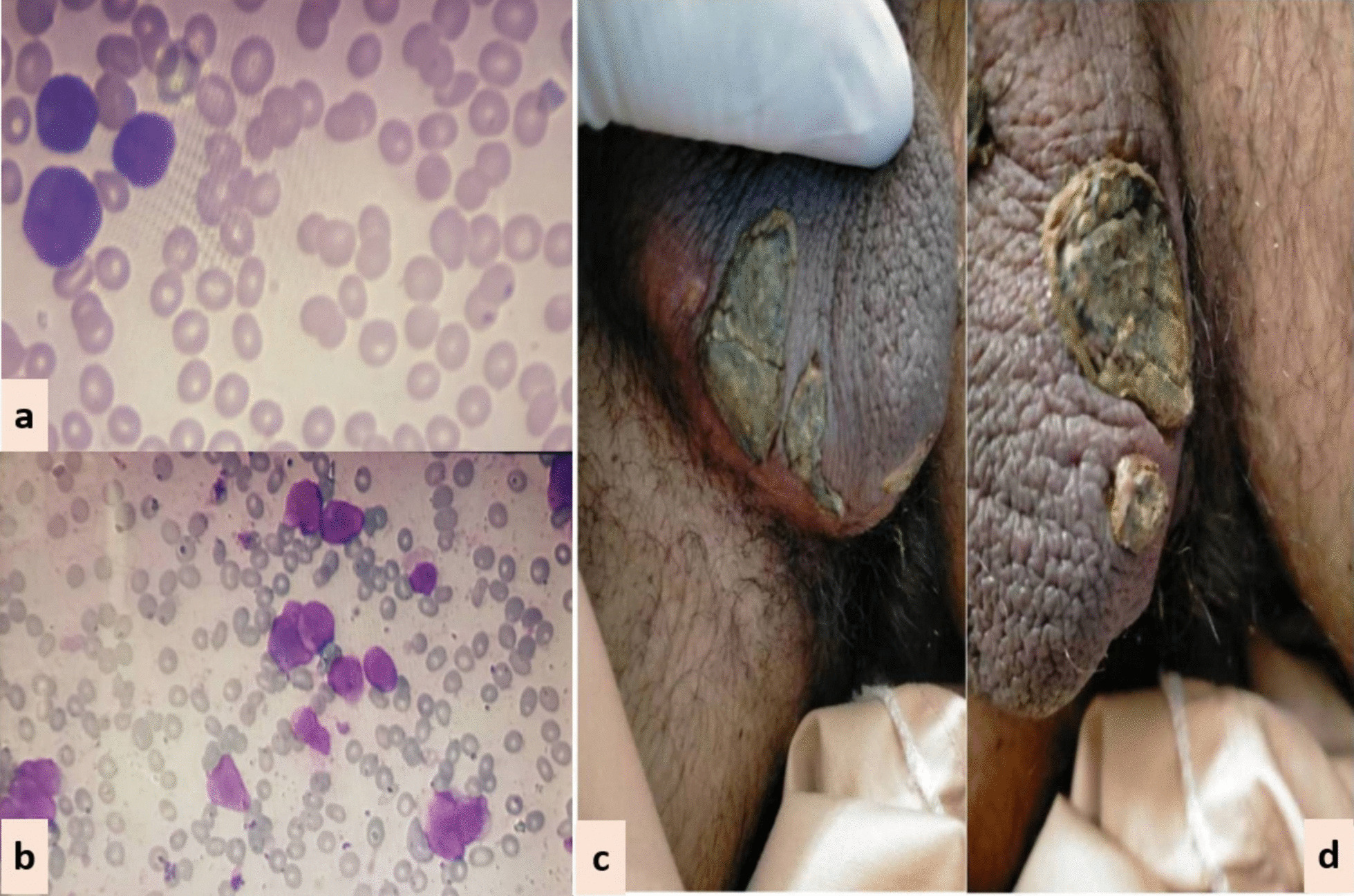
Scrotal lesions (CASE 3). **a** Peripheral smear. **b** Bone marrow aspirate. **c** and **d** Necrotic scrotal lesions with black eschar

In all three patients, ATRA was started as soon as abnormal promyelocytes were documented on peripheral smear/bone marrow aspirate examination (Figs. 1, 2, 3). Diagnosis was further confirmed through cytogenetic analysis as well as PML-RARA detection by polymerase chain reaction. Additionally, in all three patients, chemotherapeutic treatment was instituted according to European APL protocol, based on their risk-group classification.

*Case 1:* A 33-year-old Asian female presented in ER with history of fever, heavy menstrual bleeding and rash all over body. Induction chemotherapy and steroid prophylaxis was promptly started to prevent differentiation syndrome (DS). On Day-10 of induction chemotherapy, she developed high grade fever, cough and shortness of breath. High-resolution computerized tomography (HRCT) showed randomly scattered discrete nodular opacities with surrounding ground glass haze in both lung fields, suggestive of invasive fungal infection (IFI). Voriconazole was immediately started along with broad-spectrum antibiotics. She improved over the following 72 hour, and was discharged from hospital on Day-17. Subsequently, she landed in emergency department on Day-23 with severe dehydration, shivering, tachycardia, generalized skin erythema and discoloration of nail beds. Intravenous fluids and broad-spectrum antibiotics were started along with application of local emollients to prevent transdermal water loss. Over the next 24-36 hour, her general condition was stabilized however; skin exfoliation continued with complete desquamation of palms and soles (Fig. 1). Dermatology was consulted, and a clinical diagnosis of onychomadesis and exfoliative dermatitis (ED) was made. A review of her clinical case demonstrated no apparent cause for ED except for a rare association with ATRA. However, considering the curative potential of ATRA, it was continued till Day-28 as per protocol. Her skin condition gradually resolved over next 10–14 days after discontinuation of ATRA. She had recurrence of similar skin condition upon re-exposure to ATRA in her consolidation chemotherapeutic cycles, however, the exfoliation was mild and patchy that responded well to good oral hydration and local skin emollients.

*Case 2:* A 28-year-old Asian male presented in the out-patient clinic with the history of generalized weakness, high-grade-fever, productive cough and bruises over body. On examination, he had multiple ecchymosis and petechiae with coarse crepitations involving right-middle and left-lower lung fields. He was promptly started on broad-spectrum antibiotics. Additionally, as per protocol, induction chemotherapy and dexamethasone prophylaxis was also instituted. His fever and cough remained unresponsive despite broad-spectrum antibiotics. Voriconazole was instituted upon the identification of IFI on HRCT findings. By day-10, coagulopathy was normalized, and clearance of abnormal promyelocytes was documented by Day-18. On Day-20, he complained of skin dryness, itching and scaling; physical examination revealed generalized xerosis and erythema (Fig. 2). Despite aggressive skin care, generalized skin exfoliation, most pronounced on palms and soles, ensued. Clinical diagnosis of ED was established after obtaining dermatological consultation. However, in view of his clinical stability, ATRA was continued. Bone marrow aspirate on Day-28 showed morphological remission. Recurrence of erythema and exfoliation was documented during consolidation phase of chemotherapy, but the condition was responsive to local emollients and oral hydration.

*Case 3:* A 15-year-old Asian male presented in the out-patient clinic with complains of high-grade-fever, muco-cutaneous bleeding and pancytopenia. On presentation, patient was febrile and had oral thrush. After sending his baseline tests he was taken on broad-spectrum antibiotics and triazole antifungal (itraconazole). After completion of induction chemotherapy, patient was discharged with bi-weekly follow-ups.On Day15, he reported two blackish, mildly tender scrotal lesions with minimal serous discharge (Fig. 3). Antibiotic cover for soft tissue infection was commenced along with local wound care with topical steroids and antibiotics. He had no sign of systemic infection/sepsis. Local bacterial & fungal cultures and serological testing for herpes simplex virus were reported negative. Despite adequate local care and optimal antibiotic support, his lesions showed no sign of healing, and two new lesions were developed. Lesion biopsy for histopathological evaluation was declined by the patient. Keeping the rare but reported occurrence of ATRA-induced scrotal ulceration and fournier's gangrene; ATRA was transiently withheld for ten days and the lesions started to regress. However, considering the indispensable role of ATRA in APL, it was reinstituted. Scrotal lesions persisted without any worsening. ATRA was stopped after completion of protocol. Complete resolution of scrotal lesions was documented over the following two weeks. Afterwards, he received two cycles of consolidation chemotherapy, but no recurrence was reported.

## Discussion and conclusion

The antineoplastic role of ATRA remains indispensable in the curative management of APL. It is considered a relatively safe drug with a well-known toxicity profile. Commonly reported adverse events include DS, pseudotumor-cerebri, hypertriglyceridemia, transaminitis, and headache. Although, mild cutaneous toxicities like muco-cutaneous xerosis, photosensitivity, rash, pruritus and sweet’s syndrome are well reported, severe dermatological toxicities are rarely reported in literature [18, 19]. In this case series, we have discussed three cases of ATRA-induced rare dermatological complications in APL.

Case 1 and 2 developed ED during remission induction phase of chemotherapy. Literature review revealed only a single reported occurrence of ATRA-induced ED in APL by YonelIpek *et al.* [4]. ED is a potentially life-threatening cutaneous manifestation that is characterized by diffuse skin erythema and scaling. Various underlying disorders can trigger its onset through a complex interplay of inflammatory cytokines and phagocytes. In contrast to our cases, the case reported by Yonel Ipek *et al.* developed xerosis in consolidation phase, which akin to our cases started after two weeks of ATRA exposure and rapidly deteriorated to generalized erythroderma and scaling. In both cases, discontinuation of ATRA resulted in complete resolution of ED.

In case 3, we have reported ATRA-induced necrotic scrotal ulceration. Literature review revealed that over the last two decades, a total of twenty cases of ATRA-induced scrotal ulceration have been reported. Histopathological evaluation of these lesions revealed atypical granulocytic infiltration, pointing towards the possible etiological role of differentiated APL cells in the pathogenesis. Most of these cases, including ours, developed genital-lesions almost after two weeks of ATRA exposure and remained unresponsive to local and systemic antibiotics. ATRA had to be halted in most of the cases to prevent progression to fournier’s gangrene [5–17].

Scattered over the span of three years and considered in isolation, it was not initially apparent to us that all three cases had one striking similarity: concomitant use of ATRA and triazole antifungals. ATRA is primary metabolized by cytochrome P450 enzyme system. Triazole antifungals are notorious for their strong inhibitory effect on CYP450 enzyme system, resulting in supra-therapeutic drug levels and toxicity [20–22].

Potentiation of serum ATRA levels by inhibition of CYP450 system was first explored by Rigas *et al.* [23]. This study reported 1.8 times higher serum concentration of ATRA with concomitant use of ketoconazole. Since then a number of cases have reported the augmentation of ATRA-induced toxicities due to this pharmacokinetic interaction. Concomitant use of ATRA and triazole antifungals that is voriconazole and posaconazole has been implicated to cause severe hypercalcemia [24–27]. Similarly, combination with fluconazole has been reported to cause severe neurotoxicity and nephrotoxicity [28, 29].

Considering the temporal association of dermatological complications with triazole antifungals in our patients, we speculate that the concomitant use of triazole antifungals inhibited the metabolism of ATRA, resulting in higher serum concentrations and markedly accentuated cutaneous toxicities. A study further strengthening our hypothesis was conducted by Kurzrock *et al.* to evaluate the maximum tolerable dose of ATRA in myelodysplastic syndrome. The study reported severe dose-limiting cutaneous toxicities, such as generalized desquamation and genital ulceration, at doses > 150 mg/m^2^/day, compared to mild xerosis and erythema in the dose range of 45–100 mg/m^2^/day. Akin to our cases, the study reported complete resolution of cutaneous toxicities within 1–2 weeks of ATRA discontinuation [30].

Another important point is the recurrence of ED in both case 1 and 2 during their consolidation chemotherapy cycles, whereas recurrent scrotal ulceration was not documented in case 3. The most likely explanation is the continuation of voriconazole as secondary prophylaxis in patients with invasive fungal infections (IFI) (case 1 and 2), whereas itraconazole was discontinued after remission induction in case 3. This once again underscores the pharmacokinetic potentiation of ATRA-induced cutaneous toxicities by triazole antifungals. An important limitation of our study is that, due to the unavailability of serum voriconazole testing, we couldn’t document serum voriconazole levels, something that could provide valuable insights into the effect of serum azole levels on the severity of cutaneous manifestations.

By highlighting this crucial pharmacokinetic interaction and its potentially severe implications, we urge our fellow oncologists to remain vigilant regarding the inhibitory effects of triazole antifungals on the metabolism of ATRA. We propose the use of a non-myelosuppressive combination of ATRA and arsenic trioxide for APL, thereby eliminating the need for prophylactic antifungals. In the case of invasive fungal infections (IFI), we recommend considering alternative classes of antifungals. However, if triazole antifungals are deemed unavoidable, we suggest close monitoring for potential side effects and implementing prophylactic measures as clinically necessary.

## Data Availability

Data sharing is not applicable to this manuscript as no datasets were generated or analyzed during the current study.

## Abbreviations

APL: Acute promyelocytic leukemia
ATRA: All-trans retinoic acid
CYP450: Cytochrome P450
DS: Differentiation syndrome
ED: Exfoliative dermatitis
HRCT: High-resolution computerized tomography
IFI: Invasive fungal infections
PML-RARA: Promyelocytic leukemia-retinoic acid receptor alpha
